# Women's health groups to improve perinatal care in rural Nepal

**DOI:** 10.1186/1471-2393-5-6

**Published:** 2005-03-16

**Authors:** Joanna Morrison, Suresh Tamang, Natasha Mesko, David Osrin, Bhim Shrestha, Madan Manandhar, Dharma Manandhar, Hilary Standing, Anthony Costello

**Affiliations:** 1 International Perinatal Care Unit, Institute of Child Health, University College, London, 30 Guilford Street London, WC1N 1EH, UK; 2 Mother and Infant Research Activities (MIRA), GPO Box 921, Kathmandu, Nepal; 3 Nepal Administrative Staff College, Kathmandu, Nepal; 4 Institute of Development Studies, Falmer, Brighton, Sussex, BN1 9RH, UK

## Abstract

**Background:**

Neonatal mortality rates are high in rural Nepal where more than 90% of deliveries are in the home. Evidence suggests that death rates can be reduced by interventions at community level. We describe an intervention which aimed to harness the power of community planning and decision making to improve maternal and newborn care in rural Nepal.

**Methods:**

The development of 111 women's groups in a population of 86 704 in Makwanpur district, Nepal is described. The groups, facilitated by local women, were the intervention component of a randomized controlled trial to reduce perinatal and neonatal mortality rates. Through participant observation and analysis of reports, we describe the implementation of this intervention: the community entry process, the facilitation of monthly meetings through a participatory action cycle of problem identification, community planning, and implementation and evaluation of strategies to tackle the identified problems.

**Results:**

In response to the needs of the group, participatory health education was added to the intervention and the women's groups developed varied strategies to tackle problems of maternal and newborn care: establishing mother and child health funds, producing clean home delivery kits and operating stretcher schemes. Close linkages with community leaders and community health workers improved strategy implementation. There were also indications of positive effects on group members and health services, and most groups remained active after 30 months.

**Conclusion:**

A large scale and potentially sustainable participatory intervention with women's groups, which focused on pregnancy, childbirth and the newborn period, resulted in innovative strategies identified by local communities to tackle perinatal care problems.

## Background

Participatory approaches to health have been advocated since the 1978 Alma Ata declaration in which the World Health Organisation emphasised the need for citizen participation in primary health care [1]. This paper details the development and implementation of a participatory project to improve perinatal care at the community level in rural Nepal.

### Community participation in health care

The vision of Alma Ata was that increasing community participation in planning and implementation would lead to more cost-effective delivery of health care and increases in service utilisation. As communities took greater ownership of services they would become more culturally acceptable and responsive to local needs. Community participation also aimed to increase self-reliance and social awareness, which would lead to better health outcomes [2-4]. Opinions differ about the extent to which participation can achieve these results, and to what degree governments and agencies have facilitated participation, but the appeal of participatory approaches remains strong. Participation may be considered as a continuum [5]. In fully participatory approaches, needs are identified by the community themselves, who then may seek external support. At the other end of the continuum, superficial participation of community representatives is sought to validate the aims of programme planners, usually already decided.

### Harnessing the strengths of participation in community based interventions

Reproductive health is an area where participatory approaches have been attempted. A structured literature search for community-based interventions focusing on perinatal health revealed no randomized, controlled trials, but two studies in developing countries which had evaluated impact on Perinatal health outcomes. The Warmi project in Bolivia, initiated by a collaboration of Save the Children Federation, USA and USAID MotherCare project [6], worked with women's groups to reduce maternal and neonatal mortality and morbidity. They used a participatory approach involving community diagnosis, planning together, implementation of plans, and participatory evaluation. The Warmi project, though neither randomized nor controlled, and based on a before-and-after analysis of 639 and 708 births, did report a reduction in the perinatal mortality rate from 117 to 44 per thousand births. The activities initiated by women's groups included literacy programmes, savings and credit schemes, and programmes to increase access to family planning.

Studies based in the community that are towards the low end of the participation continuum also appear to have been successful in enabling improvements in pregnancy outcomes. A study in Maharashtra state, India, tested the effectiveness of early detection of warning signs of illness and village level management of neonatal sepsis (a cause of many neonatal deaths in developing countries)[7]. Village health workers were trained to visit newborn infants in their homes and identify and treat neonatal sepsis. This intervention appeared highly successful as a drop in neonatal mortality of 62% occurred. Village health workers were intensively managed and supported by the research team, and therefore large-scale implementation may be difficult. The study did, however, provide evidence that community level interventions to prevent or treat problems of the perinatal period in developing countries could be cost-effective.

### The Nepal MIRA Makwanpur trial

The MIRA Makwanpur trial was designed to test the impact on neonatal mortality of a participatory intervention with women's groups, based on the Warmi Bolivia model, but on a much larger scale and using a randomized and controlled design. In south Asia infant mortality rates fell steadily from 1970 to 1990, but the decline has subsequently plateaued. In order to reduce infant mortality rates further, a focus on the neonatal period, in which most infant deaths occur, is necessary [8,9]. Primary and secondary care are deficient in rural areas of Nepal and where services exist, the reasons for their underuse are complex. The topographical barriers combined with limited expenditure on public health, poor quality of care, a high turnover of service providers, a lack of drugs and supplies and a lack of ownership of health programmes by communities all contribute to issues of demand and supply.

The trial was implemented by a Nepali non-governmental organization, MIRA (Mother & Infant Research Activities). MIRA has been working in Nepal since 1992, conducting research specifically about newborn care, and is headed by a senior pediatrician (DM). The trial involved 24 Village Development Committees in rural Makwanpur district. Ethical approval was sought from the Nepal Health Research Council, and local meetings were held with the District Development Office and Chief District Officer to discuss the aims and objectives of the study. The chairpersons for each Village Development Committee agreed to take part in the study and provided signed consent, and links were made with community leaders, district health services and non governmental organisations. Each Village Development Committee has an average population of 7000 (range 1576 to 23 429) divided between nine wards. In twelve of the Village Development Committees a trained, locally based facilitator was employed to mobilize women's groups. All pregnancies and births to married women of reproductive age were monitored in the community. Details of the monitoring and the design of the trial have been described elsewhere [10] and the effect of this intervention on birth outcomes was reported in a recent publication [11]. Astonishingly, there was a reduction in neonatal mortality by 30% in intervention clusters, and an even larger and statistically significant effect on maternal mortality rates (78% reduction), although caution is required in interpretation given the relatively few maternal deaths. This paper describes and analyses the implementation of the first stages of the participatory intervention over a 30 month period.

## Methods

### Setting of intervention

Nepal has a population of 23 million and a per capita gross national product of 240 US dollars. Literacy rates have improved steadily, particularly for females (currently 43%), but there remain gender disparities in literacy, school enrolment, and school dropout rates [12].

Life expectancy is now 61 years[13]. The total fertility rate is 4.1, the under-five mortality rate 91, the infant mortality rate 64, the neonatal mortality rate 39 per thousand live births, and the perinatal mortality rate 47 per thousand births [9]. The maternal mortality ratio is estimated at 539 per 100 000 live births [14]. Access to health care is limited as a result of geography, limited expenditure on public health, variable quality of care, high turnover of service providers, a lack of drugs and supplies, and lack of ownership of health programmes by communities.

Makwanpur district, south west of Kathmandu, has a population of 376 000 [15] and a Human Development Index of 0.31, close to the national median. Makwanpur comprises hill and plain areas, with 15 different ethnic groups, the largest being Tamangs, a Tibeto-Burman group. Data from our baseline survey showed that more than 90% births take place at home and only five percent are attended by a trained birth attendant. The first health care provider in times of maternal or neonatal illness is the shaman (*dhami jhankri*) or traditional healer [16].

### The intervention process

The first ten meetings of the women's group participatory intervention were based on the design of the Warmi project in Bolivia [17]. In order to enter the communities successfully we gathered detailed information on local social networks and organizations, as well as attitudes and practice around the time of pregnancy and birth. Social mapping and qualitative research were conducted and served as a training exercise in facilitating focus group discussions and building rapport in the community [18]

### Establishing facilitated women's groups

Meetings were facilitated by a paid, locally based woman, who was selected on merit and trained in facilitation techniques. The position of facilitator was locally advertised and suitable candidates were interviewed by senior MIRA employees. Each facilitator is paid a salary slightly higher than the government equivalent (5330 Nepalese rupees, or 71 US dollars). Her full-time responsibility was to plan and facilitate monthly women's group meetings, each facilitator leading nine groups per month, covering an average population of 7000. Meetings were organized in co-ordination with the local Female Community Health Volunteer, an unpaid community based health worker. In profiling our study area, we found that nongovernmental organisations or community based organisations did not routinely work in all 24 of our study Village Development Committees and had different agendas. The female community health volunteer works at ward level, and as part of her job description she runs women's groups to conduct health promotion activities.

**Figure 1:**
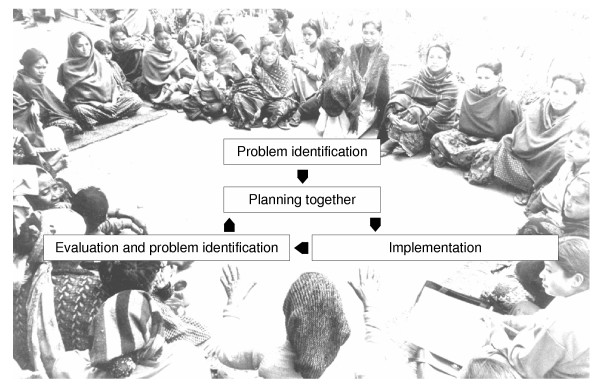
The community action cycle

The facilitator used a meeting manual, adapted from the Warmi project, to guide the women's groups through problem identification and community planning using participatory iterative methods (see Figure 1 and Table 1). Facilitators were trained in the use of this manual and were allowed scope for their own input. Facilitation supervisors were also appointed after national advertisement and formal interview, and two men and three women were selected. One supervisor was provided for every three facilitators, providing support through community visits and regular meetings.

**Table 1:** Content of first ten women's group meetings

Stage in the cycle	Meeting	Content
Introduction	1	To introduce the group to MIRA Makwanpur's work
	2	To discuss why mothers and newborn infants die To introduce how MIRA will work in the community
Problem	3	To find out how women understand maternal and neonatal problems
Identification	4	To find out what kind of maternal and neonatal problems are in the community
	5	To discuss whether the maternal and neonatal problems are common To identify strategies to collect information from the community
Problem Prioritisation	6	To share the information collected from other women in the community To decide what are the three most important maternal and neonatal health problems
Planning together	7	To discuss possible strategies for addressing the prioritised problems
	8	To discuss which other community members should be involved in developing strategies
	9	To discuss how to prepare for the community members meeting
	10	The community members will learn about what the women have been doing The community members will learn about the three problems identified by the women The community members will learn about the possible strategies To reach a consensus of the strategies

#### First meetings and problem identification

**Figure 2:**
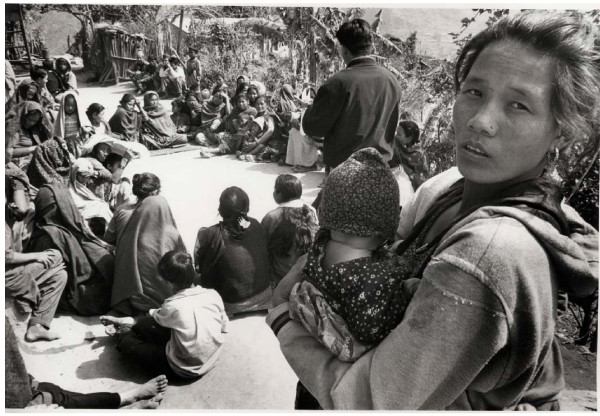
Women's group and facilitation manager

Facilitators and supervisors were responsible for creating awareness and interest in their communities about the meetings, and in most wards at least 20 women attended the first few meetings (see Figure 2). Time was taken to introduce the study agenda to the groups, especially important in areas where many non governmental organisations work and expectations were often high. The first three meetings facilitated discussion of the reasons why mothers and newborn infants die in the community. The reasons for death were discussed in terms of social as well as medical factors with the aid of a story [19]. Women were introduced to the concept of '*learning together*' through another story, and were encouraged to discuss perinatal problems within the group and with their neighbours and friends. In this way the facilitator and the women learned which perinatal problems affected their community. Each group prioritised three problems of newborn infants and/or pregnancy which were recorded with justification for their inclusion. Most group members were illiterate and therefore facilitators used pictures and voting with stones to prioritise problems.

#### Planning together

The objective of these meetings was to encourage women to identify local and low cost ways of tackling the prioritised problems using local resources. Many examples were listed in the manual and the supervisor was encouraged to support the facilitator during these meetings. The idea behind these meetings was to enable the women to prepare a plan to tackle the problems they had found, which would then be presented to their community.

#### The community meeting

A community meeting was planned and organized by the groups to enable increased community participation and to legitimize the work of the group. The community was invited to hear what the women's group had been doing, and to participate in *planning together *strategies. Most groups decided that community leaders would be invited by letter, and other households would be verbally invited. The groups also discussed the way they would present their findings and practised to develop their confidence.

The supervisor supported the local facilitator and the group, and played a key role in facilitating the meeting. After introducing MIRA and its role in the community, the women's group presented their prioritised problems and suggested strategies to tackle them.

#### Methods of data collection and analysis

Data were collected through a variety of qualitative techniques. Participant observation was carried out by the technical advisors to MIRA (NM and JM). They helped design and implement the intervention, and lived in the vicinity of the head office in Hetauda, Nepal, throughout the study period (JM succeeded NM). They visited the field many times and attended facilitation team meetings regularly. The advisors are of British nationality but have an excellent spoken command of Nepali language and have a background in anthropology and sociology. Although they did not keep a diary as is usual in participant observation, their reflections and observations were noted in monthly reports and topic reports which have been used in this analysis. These reports were also contributed to and discussed with the facilitation team (facilitators, supervisors, and senior facilitation manager) who also added their reflections and observations. The technical advisor (JM) and senior facilitation manager (ST) analysed monthly reports and meeting minutes and reached consensus on themes emerging from the data, and issues of interest. Although there are limitations to this method of data analysis (the cultural background of the technical advisors and the fact that a diary was not kept), we wished to present the results of operational research that makes use of less formally recognized qualitative data collection techniques. Analysis of the data by more than one person strengthens the analysis and using different methods of data collection enables triangulation of the data.

## Results

Out of 111 women's groups, 77 moved on to develop and implement strategies and 100 groups continue to meet to discuss perinatal health. Particular reasons for which the remaining groups did not meet include the unstable security situation, lack of support from local leaders, husbands or health workers, and general lack of interest.

### What makes an active women's group?

The continuing activity of most groups suggests that usually group members found the experience useful and enjoyable. Not surprisingly, the activity of the groups varied. We found no specific formula for an active women's group. Previous studies suggested that homogeneity of members was conducive to a successful group [20,21], but our groups showed much ethnic and social diversity. Issues of ethnicity, geography and distance from a market area did not uniformly affect the activity of groups. For example, there were two particularly active groups near market areas, but in other areas factors concurrent with living near a market – such as higher socioeconomic status and less cohesion between households – did not facilitate enthusiastic women's groups. Some groups were dominated by women from higher castes, but in others these higher castes served as a stimulant to more traditionally subservient or timid ethnic groups. Issues of local support from political groups, local health staff and men also seemed important. Other studies have also found that supportive husbands make it easier for women to participate in groups [22]. In most communities supervisors and facilitators had been successful in establishing good community rapport, and strategies were agreed to help maintain community support, such as facilitators attending antenatal clinics and supervisors presenting reports to Village Development Committee chairpersons.

### Problem prioritization

Women actively participated in learning together and gathered much information from their communities. The prioritized problems reflected local perceptions of the seriousness and frequency of specific problems and hence were different in each community (See Table 2).

**Table 2:** Problems prioritised by women's groups

Neonatal Problems	Number of groups
Pneumonia in the newborn infant	31
Low birth weight	16
Jaundice in the newborn infant	12
Neonatal death	7
Breathing problem in the newborn infant	6
Infant not feeding	4
Green stool in the newborn infant	3
Wounds in the newborn infant	2
Tetanus in the newborn infant	1
Eye and ear infection in the newborn infant	1
	

Maternal problems	

Retained placenta	58
Vaginal Discharge	42
Malpresentation	30
Headache in the mother	22
Post partum haemorrhage	20
Fever (unspecified if in mother or baby)	15
Breast problems	13
Ante partum haemorrhage	11
Miscarriage	11
Abdominal pain in the mother	6
Oedema of hands and legs in the mother	6
Prolonged labour	5
Maternal death (delivery complications)	2
Anaemia in the mother	2
Vomiting in the mother	1
Missing data	3

Total	330

### Planning together

During the community planning meeting groups nominated members to present their findings and eight groups performed small socio-dramas. When local health personnel and area chairmen attended, discussions were livelier and planning more productive. Issues of health care underutilization by the community or issues of poor service delivery were often raised. In nine places, communities appeared apathetic towards the group and were not prepared to commit or participate in planning. In these instances, they were happy for the group to plan and implement strategies and little discussion took place. In four places the group met with hostility from community leaders or health personnel or exceptionally low attendance from the community, usually due to local grievances with staff selection procedures or to the unstable security situation.

### Strategy development and implementation

The strategies that were discussed during *planning together *and have been most successfully implemented were the mother and child health fund, locally produced clean home delivery kits, management and production of stretchers, and awareness raising through video shows.

#### Mother and child health fund schemes

69 groups favoured mother and child health funds as a way of overcoming the cost barriers to seeking and obtaining care. The cost of consultation, medicine and transport is a real reason that families do not gain access to services in Nepal [23]. MIRA provided training to fund management committee members elected from each group. These committees sometimes included literate community members not attending the group. Each group developed their own policy with regard to how money would be collected, who would be able to access it, how often it would be collected, and who would be responsible for managing it. 23 months after the first mother and child health fund was established, groups had generated between 731 rupees (10.5 US dollars) and 9635 rupees (133.8 US dollars).

#### Clean home delivery kits

The clean home delivery kit is advocated by the World Health Organisation as an effective way to promote cleanliness during home delivery and to reduce the risk of maternal and neonatal infection [24,25]. In Nepal, a local private company (MCH Products Pvt Ltd) has produced a clean home delivery kit, approved by the Ministry of Health, which contains a blade, a bar of soap, three cord ties, a plastic coin for cord cutting, a plastic sheet, and a set of pictorial instructions. There are problems with distribution to remote rural areas and other difficulties regarding local acceptability and price [26].

A few groups were keen to develop their own locally produced clean home delivery kit, and facilitators disseminated this idea to motivate other groups by example. 19 groups have made clean home delivery kits and four groups have reproduced subsequent batches. Groups have decided the price, but all groups sell at a lower price than the MCH products kit, with profits going into the mother and child health fund. The pictorial instruction leaflet was developed with a local artist and was piloted in the community. The groups have also explored different selling points: local shops, Female Community Health Volunteers and Traditional Birth Attendants, and group members sold kits to their friends and neighbours. Recently, groups from one Village Development Committee have used free distribution of clean home delivery kits as an incentive to attend for antenatal care; the kits are free for women who attend at least four times.

#### Stretchers

In the study area, most births take place in the home [27] and transportation of women who encounter problems is difficult. Women's groups therefore identified the need for stretchers. 19 groups decided to raise money for stretchers themselves and the other 23 groups utilized local resources such as forest user groups or Village Development Committee offices. Women's groups investigated if there were any existing stretchers in their area, or if these needed repair. One group felt that the modern style of stretcher was not suitable for carrying women across difficult terrain, and therefore made a bamboo basket (*dhoko*) which is traditionally used to carry fodder or crops using a head strap (*tump line*). Some women's groups assumed management of unused stretchers, ensuring their accessibility and promoting their use, with 35 groups levying a fee for usage

#### Awareness raising through video shows

During the community meetings, many groups felt that there was a lack of awareness about perinatal health problems and how to deal with them. MIRA had previously researched and produced a 20 minute film about newborn care in Makwanpur and the groups were keen to use it in their communities. Group members approached those households in the community with electricity and a television, and the video was shown in homes or public buildings. Although not all of the study area has electricity, the video was shown in 10 out of 12 Village Development Committee areas, attracting an audience of more than 2100.

### Participatory health education

During the identification of strategies to address problems, there was a tendency to mismatch prioritized problems and strategies. For example, one group suggested tackling the problem of post-partum hemorrhage by attending antenatal care. Another group considered that the problem of vaginal discharge during pregnancy could be addressed by training new Traditional Birth Attendants. During the first ten meetings, and from previous data analyses, the team found an overwhelming preference for care within the community, in terms of place of birth and seeking solutions to health problems [16,27]. Home practices with unequivocal allopathic clinical benefit were rarely mentioned. There was also little knowledge about what kind of problems could be managed at different health service institutions, and it appeared that communities define the "seriousness" of a problem in a different manner to the allopathic model.

Therefore, the team concluded that perinatal health education would be useful during the development of the strategies. It was felt important to avoid turning the facilitators into educators, and therefore a participatory form of health education was developed, based around a picture card game.

#### The picture card game

**Figure 3:**
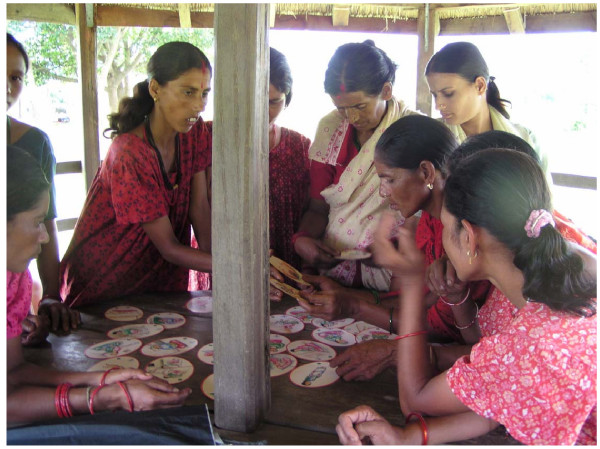
Women's group using picture cards

**Figure 4:**
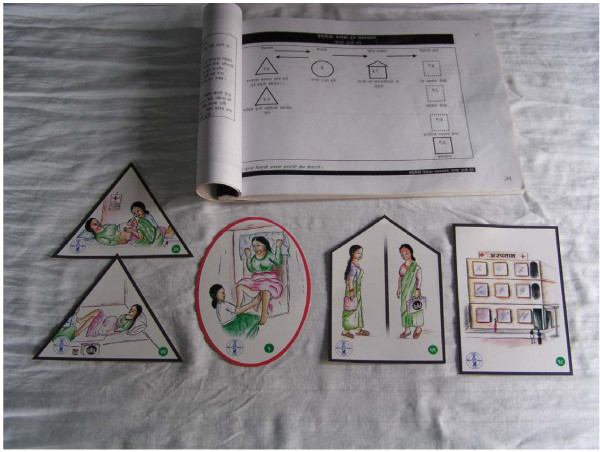
Picture card game and manual

A packet of small hand held cards of different shapes was developed in order to address the mismatch between problems and strategies and to promote participatory learning. Each shape of card represents either a problem (circle shape), a prevention activity (triangle shape), a home-care activity (house shape), or a health institution (square shape). The cards are pictorial and were developed with the MIRA health team and a local artist (see Figures 3 and 4). They were extensively piloted with women's groups and adapted accordingly. A manual for facilitators was also developed to accompany the cards. The card game is played in the group by passing round the cards and discussing the pictures. The group members match problem cards to their corresponding prevention activities, home care activities or type of health institution that could treat that problem. The card game worked well in facilitating discussion, and women and facilitators both enjoyed the learning experience. The team completed a participatory evaluation of the game with a sample of groups which indicated that the game also facilitated learning about danger signs, home care and prevention activities. Group members are presently taking the picture cards on visits to pregnant women in the community who are not group members.

### Service quality spin-offs

#### Community health volunteers

The facilitator has worked to involve and support female community health volunteers with their work in the community. 70 group meetings have regular attendance and active involvement of the local female community health volunteer and traditional birth attendant. The female community health volunteer is the lowest cadre of government appointed health staff and is responsible for one ward. She is unpaid and has a broad job description mainly focused around health education. In theory, she should run monthly women's group meetings to facilitate health education and discuss issues of maternal and newborn health. Although she receives initial and refresher training, she is often left to work unsupervised and unsupported, and in practice female community health volunteers find it difficult to run women's groups. By seeking the participation of the female community health volunteer in the groups, we gave her a forum to conduct her work and increase her contact with her user group.

In twelve wards the women's group was invited by the local health institution to play an active role in selection of new female community health volunteers and traditional birth attendants. The group had created good links with the health institution, which could be exploited for future service quality improvements. Clearly, the health institution and the community consider many groups as legitimate entities with a role to play in the health of women and their children. It also appears that members of women's groups have become more involved with their local health services.

In one area women's group members responded to the needs of women visiting an outreach clinic for antenatal care and family planning. Women were complaining of a lack of privacy and there was no furniture in the clinic. The group contacted the local forest user group to supply materials for rudimentary furniture and collected money for the purchase of cloth for curtains. In several cases, the women's group was a medium for brokered links between health service providers and users.

### Proxies for empowerment effects?

One group put a sign on the door of their meeting place indicating a sense of ownership. Ten out of 12 facilitators have been invited to participate in other meetings and community activities as their role as key actors in the community is being recognized and developed. Women's groups have sung the song from the video film at the annual women's festival, and a supervisor initiated discussion about newborn health in a local bus using a cassette of the song from the film. Another women's group organised a perinatal care quiz that was carried out with nearby women's groups and community members.

## Discussion

We have described the development and implementation of a large scale participatory intervention with women's groups. This was adapted from a smaller scale project developed in Bolivia, and implemented in a poor rural population of 86 000 in Nepal. The effects on health outcomes, reported elsewhere, were dramatic.

During the process of developing and implementing the intervention we had to be flexible and respond to the needs of the group. Group members and the wider community clearly faced difficulties when thinking about ways to tackle perinatal problems. These difficulties raised issues around culture and our facilitation role.

### Striking a balance between support and directiveness

It is highly likely that the facilitation team's attempts to adequately support the facilitators may have led to less participatory processes taking place, especially in the case of strategy development. The facilitation manual was considered by the facilitation team as an essential resource. Examples were often given to enable facilitators to grasp key concepts before conducting a meeting. The manual was designed as a reference guide but evidently became more like an instruction booklet, as the strategies most commonly adopted during community meetings were those given as examples in the manual. The reasons behind this usage of the manual illustrate some of the key issues in implementing a participatory project. To truly facilitate, and not be directive, is a difficult technique to learn, especially in a hierarchical society where the facilitator's education has emphasised rote learning rather than independence of thought [28]. Our facilitators were also keen to educate and provide the answers, and it may have appeared easier (in the short term) to suggest things for groups to do than to facilitate open discussion. The self-confidence or ability of the facilitator to manage the chaos and unpredictability resulting from a truly participatory process was often lacking, although their facilitation skills developed with time.

### Power and culture

Difficulties in linking problems to strategies may also be explained in part by the cultural phenomenon of fatalism, '*ke garne' *(*what to do*?). Bista described '*ke garne*' as a belief in fatalism which leads to the feeling that "*one has no personal control over one's life circumstances, which are determined through a divine or powerful external agency*" [29]. He argued that this fatalism and dependency affects the work ethic and achievement motivation in Nepal. Concepts of planning, orientation to the future, and sense of causality, are all affected. Our study experience was that fatalism affected both the way people viewed themselves in relation to a problem, and also the power and capacity they believed themselves to have in overcoming it.

### What has been the impact of the women's groups and their strategies?

In cases where there was a mismatch between problem and strategy, or when groups developed strategies suggested by MIRA, we hope that these groups will benefit from the implementation process alone. The strategies in the manual are not necessarily evidence-based, and it may be that the process of implementation is more beneficial than the strategy itself. Through implementation, interaction between the wider community and the group may be increased, knowledge about the group may spread, and more people may become interested and involved in issues of perinatal health. To enable a better understanding of the intervention process, evaluations using both qualitative and quantitative methodologies are underway. The impact of the women's group intervention was evaluated in a cluster randomized controlled trial which showed a 30% reduction in neonatal mortality rates and a reduction in maternal mortality rates in the first 30 months of the trial[11]. Qualitative analysis will explore perceptions and process indicators to assess how the intervention affected the study area and community stakeholders. Cost analysis of the intervention will enable estimates of cost-effectiveness and sustainability to be made. A comparison of the socio-economic status of women's group members with non-group members will allow an estimate of the equitability of the intervention.

The Millennium Development Goals for reductions in maternal and neonatal mortality in developing countries are unlikely to be met by 2015. In populations where maternal and newborn mortality rates are highest, most deliveries occur at home. It is essential that Safer Motherhood and Newborn Care Programmes design interventions which reach out to the poorest groups in order to change care practices at home, and care seeking for illness or complications of childbirth. Our participatory work with women's groups provides a model for an intervention that can be scaled rapidly in even the poorest and most remote communities.

## Conclusion

A large scale participatory intervention to improve pregnancy outcomes in rural Nepal through 111 women's groups has been described. Although we have faced contextual, cultural and security problems, we believe that the participatory approach can be a powerful tool in unleashing the creative potential to solve perinatal health problems in communities. Such an approach may have lasting benefits, affecting behaviour in subsequent pregnancies.

## Competing interests

The author(s) declare that they have no competing interests.

## Contribution by authors

JM wrote the first draft of the paper and contributed to the study design and collection of field data. ST, and BS contributed to the study design, collection of field data and analysis, and criticised later drafts of the paper. NM and DO contributed to the study design and analysis, and criticised later drafts of the paper. MM and HS contributed to the design of the study and criticised drafts of the paper. DM and AC contributed to the design of the study and supervision of the field programme, and criticised drafts of the paper. JM and AC will act as guarantors for the paper.

## Pre-publication history

The pre-publication history for this paper can be accessed here:


